# Ablation of somatostatin cells leads to impaired pancreatic islet function and neonatal death in rodents

**DOI:** 10.1038/s41419-018-0741-4

**Published:** 2018-06-07

**Authors:** Na Li, Zhao Yang, Qing Li, Zhen Yu, Xu Chen, Jia-Cheng Li, Bo Li, Shang-Lei Ning, Min Cui, Jin-Peng Sun, Xiao Yu

**Affiliations:** 1 Key Laboratory of Experimental Teratology of the Ministry of Education and Department of Physiology, Shandong University School of Basic Medical Sciences, Jinan, Shandong 250012 China; 2 Department of Biochemistry and Molecular Biology, Shandong University School of Basic Medical Sciences, Jinan, Shandong 250012 China; 3 Department of General Surgery, Qilu Hospital Affiliated to Shandong University, Jinan, Shandong 250012 China; 4 School of Medicine, Duke University, Durham, North Carolina 27705 USA

## Abstract

The somatostatin (SST)-secreting cells were mainly distributed in the pancreatic islets, brain, stomach and intestine in mammals and have many physiological functions. In particular, the SST-secreting δ cell is the third most common cell type in the islets of Langerhans. Recent studies have suggested that dysregulation of paracrine interaction between the pancreatic δ cells and β cells results in impaired glucose homeostasis and contributes to diabetes development. However, direct evidence of the functional importance of SST cells in glucose homeostasis control is still lacking. In the present study, we specifically ablated SST-secreting cells by crossing *Sst-cre* transgenic mice with *R26*^*DTA*^ mice (*Sst*^*Cre*^ *R26*^*DTA*^). The *Sst*^*Cre*^ *R26*^*DTA*^ mice exhibited neonatal death. The life spans of these mice with severe hypoglycemia were extended by glucose supplementation. Moreover, we observed that SST cells deficiency led to increased insulin content and excessive insulin release, which might contribute to the observed hypoglycemia. Unexpectedly, although SST is critical for the regulation of insulin content, factors other than SST that are produced by pancreatic δ cells via their endogenous corticotropin-releasing hormone receptor 2 (CRHR2) activity play the main roles in maintaining normal insulin release, as well as neonatal glucose homeostasis in the resting state. Taken together, our results identified that the SST cells in neonatal mouse played critical role in control of insulin release and normal islet function. Moreover, we provided direct in vivo evidence of the functional importance of the SST cells, which are essential for neonatal survival and the maintenance of glucose homeostasis.

## Introduction

The maintenance of blood glucose homeostasis is critical for many physiological processes, which are tightly regulated by the concerted actions of hormones, such as glucocorticoids, epinephrine produced by the adrenal glands, and insulin and glucagon generated in pancreatic islets. Although many hormones increase the glucose level in blood and exert mutual compensatory effects, insulin is the only blood glucose-lowering hormone that is indispensable for maintaining regular blood glucose levels, indicating an essential role of pancreatic islet homeostasis in blood glucose control. Accordingly, dysregulation of insulin and glucagon secretion induced by genetic, epigenetic, or environmental factors has been reported in severe metabolic syndrome^1–3^. For example, an early-onset loss of pancreatic α cells and a concomitant increase in β cells is observed in mice carrying an *aristaless-related homeobox* (*Arx*) mutation, resulting in severe hypoglycemia and neonatal death^4^. In contrast, mice with *paired box gene 4* (*Pax4*) deficiency exhibit the opposite phenotypic changes, showing both dramatic reduction in β cells and hyperglycemia-induced early death^5^. Moreover, mice lacking both the *Arx* and the *Pax4* genes display significant shrinkage of both α- and β-cell lineages and die neonatally because of lethal hyperglycemia^6^. These findings suggest the importance of the composition and architecture of islets in maintaining the necessary glucose homeostasis in neonatal mammals.

In addition to glucagon-secreting α cells and insulin-secreting β cells, the islets contain at least three other types of endocrine cells, including somatostatin (SST)-producing δ cells, pancreatic polypeptide-producing pp cells, and ghrelin-producing ε cells. The pancreatic δ cells, which release SST, regulate glucagon and insulin release in a paracrine manner^7^. Impaired release of SST from δ cells results in compromised paracrine control of β-cell activities, contributing to the pathogenesis of diabetes mellitus^8,9^. Conversely, inappropriately increased SST secretion impairs islet homeostasis and glucose tolerance^10^. However, despite the progress in this research field, the functional importance of SST-secreting cells remains elusive. Notably, whereas *somatostatin* gene knockout mice display increased glucagon and insulin release in response to nutrient stimuli compared with control mice, they show similar growth curves, islet sizes, hormone contents, resting normoglycemia and insulin sensitivity^7,11^. These observations imply that SST-producing cells may be dispensable for resting blood glucose control.

In the present work, we generated *Sst*^*Cre*^ *R26*^*DTA*^ mice, in which the SST-producing cells, including but not limited to those in the pancreatic islets, stomach, brain and intestine were specifically ablated via DTA expression. These mice exhibited disturbed blood glucose homeostasis and died within 24 h. The life expectancy of these mice with severe hypoglycemia was increased after glucose supplementation. We demonstrated that SST cell ablation directly induced proportional changes in several types of hormone-producing endocrine cells within the islets and caused excessive insulin synthesis and release, which might contributed to the hypoglycemia. Further mechanistic analyses suggested that basal insulin release in neonatal mammals is regulated by pancreatic SST-producing δ cells through a SSTR-independent but corticotropin-releasing hormone receptor 2 (CRHR2)-dependent pathway.

## Results

### SST cell ablation induces neonatal death and severe hypoglycemia

**Fig. 1:**
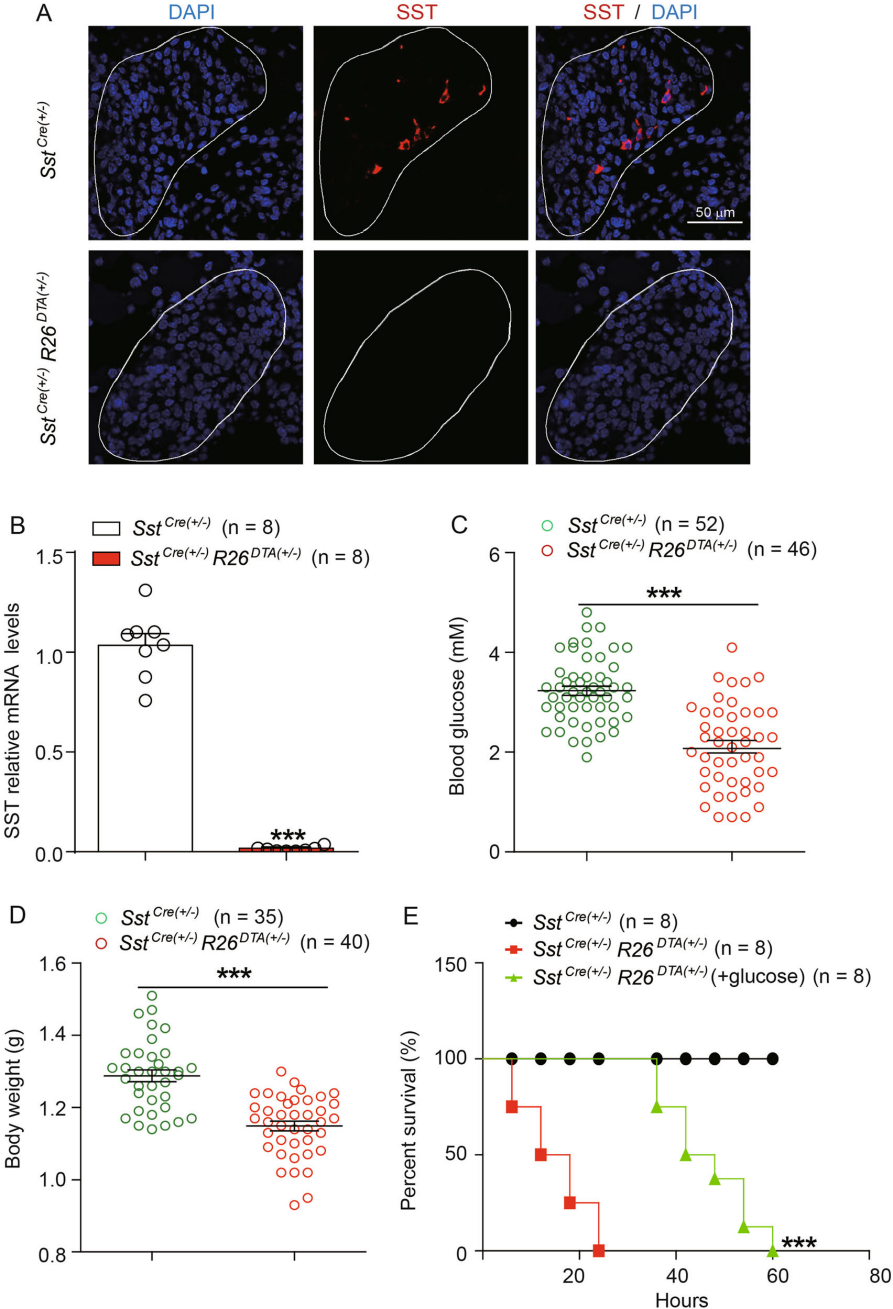
SST cell ablation induces neonatal death and impaired glucose homeostasis. **a** A representative immunostaining result for somatostatin (SST, red) in pancreatic sections from neonatal *Sst*^*Cre*^ and *Sst*^*Cre*^*R26*^*DTA*^ mice (scale bar: 50 µm). The localization of SST in SST-secreting cells was absent in *Sst*^*Cre*^ *R26*^*DTA*^ mice, conforming the deficiency of pancreatic SST-secreting cells. **b** The mRNA levels of SST in the pancreas of neonatal *Sst*^*Cre*^ and *Sst*^*Cre*^ *R26*^*DTA*^ mice determined by qRT-PCR (*n* = 8). **c,** **d** The blood glucose levels (**c**) and body weights (**d**) of neonatal *Sst*^*Cre*^ and *Sst*^*Cre*^ *R26*^*DTA*^ mice. *n* = 35–52 mice per group. **e** Survival curves comparing survival rates of *Sst*^*Cre*^ mice, *Sst*^*Cre*^ *R26*^*DTA*^ mice, and *Sst*^*Cre*^ *R26*^*DTA*^ mice treated with glucose (*n* = 8 mice per group). **b**-**d** ***p < 0.001; *Sst*^*Cre*^ *R26*^*DTA*^ mice were compared with their *Sst*^*Cre*^ littermates. Data were shown as mean ± SEM. The data statistics were analyzed using one-way ANOVA. **e** ***p < 0.001; *Sst*^*Cre*^ *R26*^*DTA*^ mice treated with glucose were compared with those treated with vehicle only. The data statistic was analyzed using log-rank test

To characterize the functional role of SST-producing cells, we generated cell-specific diphtheria toxin A chain (DTA)-expressing mice (*Sst*^*Cre*^ *R26*^*DTA*^) by crossing *Sst-cre* transgenic mice with *R26*^*DTA*^ mice^12^ (Figure S1A). Cre recombinase, expressed under the control of the *Sst* promoter, was expected to trigger DTA expression in SST-producing cells, leading to cell-specific ablation (Figure S1B-C). Immunofluorescence and quantitative reverse-transcriptase PCR (qRT-PCR) analyses confirmed that the expression of SST in the pancreatic islets, stomach and brain of the *Sst*^*Cre*^ *R26*^*DTA*^ mice was abrogated compared with the expression in their *Sst-cre* littermates (Figs. 1a, b, Figure S2A-C).

Although the *Sst*^*Cre*^ *R26*^*DTA*^ mice appeared normal at birth, they displayed significantly reduced blood glucose levels and body weights compared with *Sst-cre* mice (Figs. 1c, d). Importantly, the *Sst*^*Cre*^ *R26*^*DTA*^ mice gradually became lethargic and died within 24-h postpartum (Fig. 1e). The observed blood glucose level of *Sst*^*Cre*^ *R26*^*DTA*^ mice was similar to the blood glucose in Arx or hepatocyte nuclear factor 3 (HNF-3)-deficient mice, with a level of ~ 2 mM (or 36 mg/dL). It is worth to note that both *Arx*^*-/-*^ mice and *HNF-3*^*-/-*^ mice were neonatal lethal^4,13^. To explore whether the lethality of *Sst*^*Cre*^ *R26*^*DTA*^ mice was related to the impairment of glucose homeostasis, 50 μl of 10% glucose was subcutaneously injected into these mice at birth and every 8 h thereafter. The applied glucose treatment showed a transient rescuing effect by extending the life span of hypoglycemic mice up to 60 h (Fig. 1e). Therefore, our results suggested that the low blood glucose might contribute to, whereas other factors played critical roles in the neonatal death in the condition of deficiency of all SST-secreting cells.

### SST cell ablation promotes pancreatic insulin synthesis and release

**Fig. 2:**
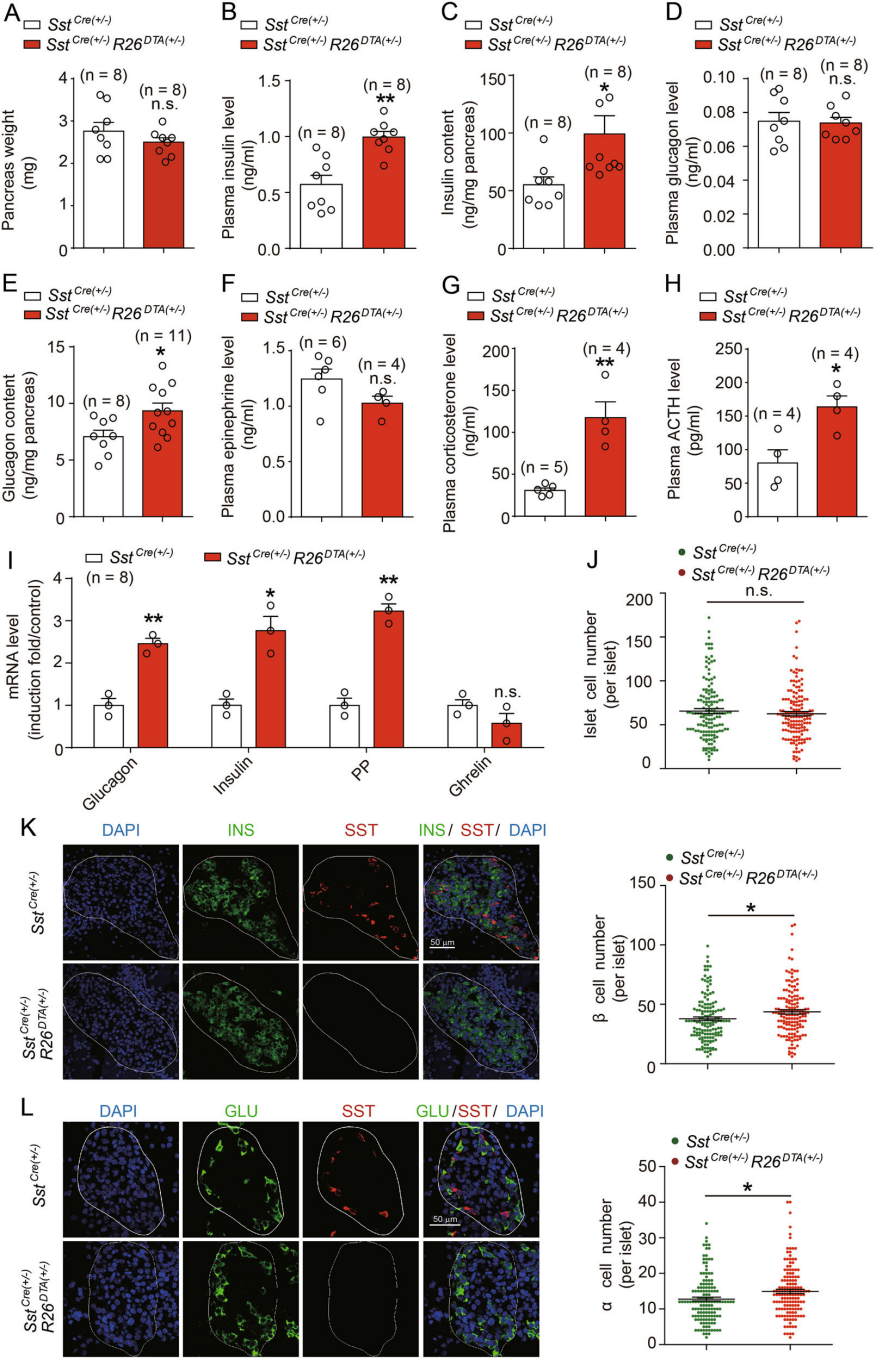
SST cell ablation leads to excessive insulin synthesis and release. **a** The pancreas weights of neonatal *Sst*^*Cre*^ and *Sst*^*Cre*^ *R26*^*DTA*^ mice. *n* = 8 mice per group. **b,** **c** The plasma insulin levels (**b**) and pancreatic insulin contents **c** of neonatal *Sst*^*Cre*^ and *Sst*^*Cre*^ *R26*^*DTA*^ mice. *n* = 8–9 mice per group. **d**, **e** The plasma glucagon levels (**d**) and pancreatic glucagon contents (**e**) of neonatal *Sst*^*Cre*^ and *Sst*^*Cre*^ *R26*^*DTA*^ mice. *n* = 8–12 mice per group. **f**-**h** The plasma epinephrine **f**, corticosterone **g**, and ACTH (**h**) levels of neonatal *Sst*^*Cre*^ and *Sst*^*Cre*^ *R26*^*DTA*^ mice. *n* = 4–6 mice per group. **i** The mRNA levels of glucagon, insulin, pancreatic polypeptide, and ghrelin in the pancreas of neonatal *Sst*^*Cre*^ and *Sst*^*Cre*^ *R26*^*DTA*^ mice determined by qRT-PCR (*n* = 8). **j** Quantification of total islet endocrine cell numbers by immunostaining. *n* = 6 mice per group. In all, 4–6 random areas were selected from each section and five sections were randomly selected from each mouse. **k** Left panel: A representative immunostaining result for somatostatin (SST, red) and insulin (INS, green) in pancreatic sections from neonatal *Sst*^*Cre*^ and *Sst*^*Cre*^ *R26*^*DTA*^ mice (scale bar: 50 µm). Right panel: quantification of islet β-cell numbers by immunostaining. *n* = 6 mice per group. In all, 4–6 random areas were selected from each section and five sections were randomly selected from each mouse. **l** Left panel: Immunostaining for somatostatin (SST, red) and glucagon (GLU, green) in pancreatic sections from neonatal *SST*^*Cre*^ and *Sst*^*Cre*^ *R26*^*DTA*^ mice (scale bar: 50 µm). Right panel: quantification of islet α-cell numbers by immunostaining. *n* = 6 mice per group. In total, 4–6 random areas were selected from each section and five sections were randomly selected from each mouse. **a**-**l** *p < 0.05; **p < 0.01; n.s. no significant difference; *Sst*^*Cre*^ *R26*^*DTA*^ mice were compared with their *Sst*^*Cre*^ littermates. Data were shown as mean ± SEM. The data statistics were analyzed using one-way ANOVA

The decreased blood glucose level observed in the *Sst*^*Cre*^ *R26*^*DTA*^ mice may be attributed to the hormonal dysregulations. These hormone may include but not limited to the glucagon and insulin produced in the pancreatic islets, the epinephrine and cortisol produced in adrenal glands. At first, we investigated the plasma levels and pancreatic islet contents of both insulin and glucagon in *Sst*^*Cre*^ *R26*^*DTA*^ and *Sst-cre* mice. Despite similar pancreas weight compared with their *Sst-cre* littermates, the *Sst*^*Cre*^ *R26*^*DTA*^ mice exhibited dramatic increases in the levels of both plasma and pancreatic insulin, by approximately twofold higher than those of *Sst-cre* mice (Figs. 2a-c). Whereas the glucagon levels in the pancreatic islets of *Sst*^*Cre*^ *R26*^*DTA*^ mice were significantly increased by approximately 20% compared with those in age-matched *Sst-cre* mice, the plasma glucagon levels of these mice were comparable (Figs. 2d, e).

We next examined the plasma levels of cortisol, epinephrine and adrenocorticotropic hormone (ACTH), as they are often involved in the counter regulatory responses to hypoglycemia. Our results revealed that whereas both the plasma corticosterone and ACTH levels in neonatal *Sst*^*Cre*^ *R26*^*DTA*^ mice were significantly higher compared with those in their *Sst-cre* littermates, the epinephrine levels of these mice were comparable (Figs. 2f-h). Because the elevated ACTH-coricosterone level should increase the plasma glucose level, the observed hypoglycemia in the neonatal *Sst*^*Cre*^ *R26*^*DTA*^ mice was not due to the over-activation of the hypothalamo–pituitary–adrenal (HPA) axis observed in these mice.

The excessive accumulation of both glucagon and insulin in the pancreatic islets of the *Sst*^*Cre*^ *R26*^*DTA*^ mice was confirmed through qRT-PCR analysis (Fig. 2i). In addition, the mRNA level of the pancreatic peptide secreted by PP cells in *Sst*^*Cre*^ *R26*^*DTA*^ mice was elevated, whereas the mRNA amount of ghrelin secreted by ε cells remained unchanged (Fig. 2i). Taken together, we observed disturbance of hormone secretion after SST cell ablation. The increase in plasma insulin together with no compensatory enhanced glucagon or epinephrine levels could be one important factor contributing to the reduction in blood glucose levels in *Sst*^*Cre*^ *R26*^*DTA*^ mice compared with those in their *Sst-cre* littermates.

The excessive synthesis of insulin and glucagon in pancreatic islets was probably due to the hyperplasia of pancreatic islets or increased proportions of hormone-secreting endocrine cells, or both. We therefore examined the number and distribution of insulin-producing β cells and glucagon-producing α cells using immunofluorescence and quantitative analysis. Notably, the total endocrine cell count in the pancreatic islets of *Sst*^*Cre*^ *R26*^*DTA*^ mice showed no significant difference compared with that of *Sst-cre* mice (Fig. 2j). In contrast, both glucagon-producing cells and insulin-secreting cells were markedly increased in the SST cell-deficient mice, which may be a compensatory mechanism for the ablation of pancreatic δ cells (Figs. 2k, l).

### SST cells regulate neonatal insulin synthesis and release through SSTR-dependent and CRHR2-dependent pathways

**Fig. 3:**
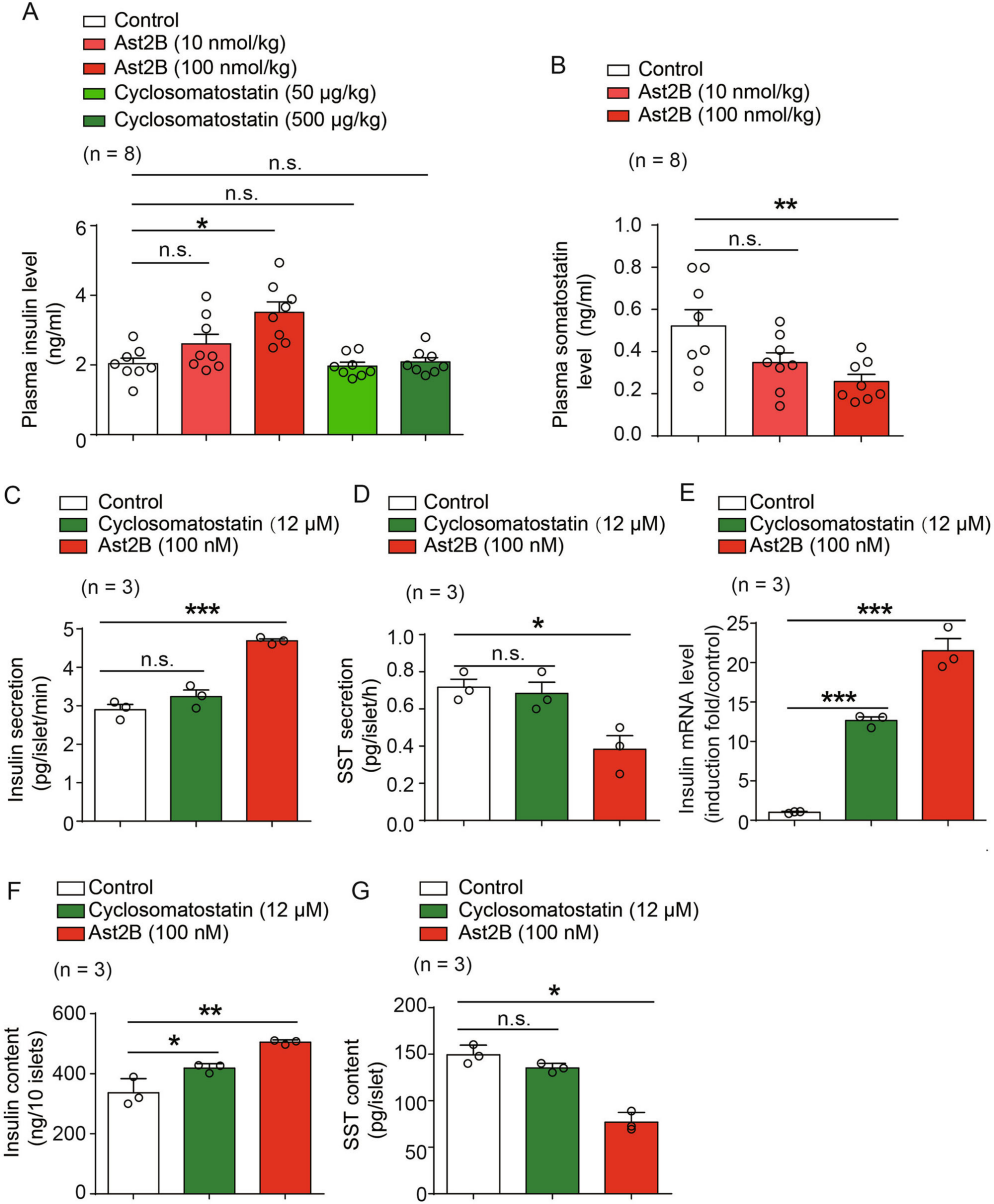
SST cells regulate insulin synthesis and release through different mechanisms. **a**, **b** Plasma insulin levels (**a**) and somatostatin levels (**b**) of neonatal rats pre-treated with SSTR antagonist cyclosomatostatin, CRHR2 antagonist Ast2B, or vehicle only (Control) for 20 min. *n* = 8 rats per group. **c**, **d** Effect of cyclosomatostatin and Ast2B on insulin release **c** and somatostatin release (**d**) of islets isolated from neonatal rats after 20 min incubation (Data are from three independent experiments. For each experiment, islets from 8 to 10 neonatal rats were picked and grouped to 50 islets for each condition). **e** Effect of cyclosomatostatin and Ast2B on the insulin mRNA levels in the islets isolated from neonatal rats after 24-h incubation (Data are from three independent experiments. For each experiment, islets from 8 to 10 neonatal rats were picked and grouped to 50 islets for each condition). **f, g** Effect of cyclosomatostatin and Ast2B on the insulin (**f**) and somatostatin contents (**g**) in the islets isolated from neonatal rats after 4-h incubation (Data are from three independent experiments. For each experiment, islets from 8 to 10 neonatal rats were picked and grouped to 50 islets for each condition). **a**–**g** *p < 0.05; **p < 0.01; ***p < 0.001; n.s. no significant difference; Rats or rats pancreatic islets treated with cyclosomatostatin or Ast2B were compared with those treated with vehicle only. Data were shown as mean ± SEM. The data statistics were analyzed using one-way ANOVA

SST is the most abundant hormone synthesized by pancreatic δ cells^14^. In general, SST exerts its function through engagement with five SST 7-transmembrane receptors (SSTR1–SSTR5), which could be blocked by the antagonist cyclosomatostatin^15^. We therefore examined whether the regulation of basal insulin synthesis and release in neonatal mammals is SST dependent via the application of cyclosomatostatin. Due to the difficulty of islets isolation from newborn mice, we alternatively chose neonatal rats for these experiments. Although the incubation of isolated neonatal islets with cyclosomatostatin significantly increased insulin transcription and insulin contents at resting state, it had an insignificant effect on basal insulin release from neonatal pancreatic islets (Figs. 3e, f). Moreover, when we directly treated neonatal rats with cyclosomatostatin at 50 or 500 µg/kg in vivo, no significant differences on the neonatal resting blood insulin level were observed (Figs. 3a-c). In contrast, incubation of cyclosomatostatin increased the glucose-induced insulin secretion (Figure S3A). These results suggest that the SST is a critical player in controlling insulin synthesis and glucose-stimulated insulin secretion, but is dispensable for the basal insulin release in resting state in neonatal mice. Factors other than the SST produced by pancreatic SST positive δ cells may play the main role in regulating the resting blood insulin level and glucose homeostasis.

We next sought an alternative approach to dissect the mechanism underlying the control of neonatal blood insulin levels by SST-secreting cells. The functions of pancreatic SST-secreting cells are tightly regulated by both the glucose level and endogenous paracrine urocortin3 (Ucn3)^8,10^. Ucn3 is co-released with insulin from β cells and triggers the secretion of SST via activating the CRHR2, limiting further insulin release from β cells. The Ucn3 receptor CRHR2 is specifically expressed in pancreatic SST-secreting δ cells, but not in other types of pancreatic islet cells, which enables the selective operation of pancreatic SST-secreting cell function using the CRHR2 antagonist astressin 2B (Ast2B)^8,10^. The application of Ast2B to isolated neonatal pancreatic islets caused decrease in the SST content and SST release (Figs. 3d-g). Moreover, direct treatment of neonatal rats with Ast2B at 100 nmol/kg in vivo resulted in significant decrease of plasma SST level (Fig. 3b). These results collectively suggest a functional role of CRHR2 in pancreatic SST-secreting δ cells in the resting state. Importantly, incubation of neonatal islets with Ast2B showed a stronger effect than cyclosomatostatin treatment on increasing insulin mRNA transcription and insulin contents (Figs. 3e, f). In particular, the inhibition of CRHR2 in pancreatic SST-secreting δ cells by Ast2B promoted insulin release from neonatal islets in the resting state, and Ast2B administration caused an increase in the blood insulin level (Figs. 3a-c). Conversely, the activation of CRHR2 by Ucn3 significantly inhibited insulin release from isolated neonatal islets at resting state (Figure S3B). Therefore, basal insulin release in neonatal pancreatic islets is regulated by SST-secreting cells through a CRHR2-mediated, but SSTR-independent pathway.

Taken together, these results reinforce the conclusion that SST-secreting cells play an indispensable role in maintaining resting blood glucose homeostasis in neonatal mammals. This regulatory process appears to be dependent on CRHR2-mediated pancreatic δ cell function, in addition to the known SST release function of δ cells.

## Discussion

As the third most common endocrine cell type in islets, the functional role of SST-producing cells has attracted intense research in recent years. For example, disruption of CRHR2 signaling in pancreatic SST-producing cells has been shown to contribute to diabetes development^8^. Furthermore, we previously demonstrated that dysregulation of the cullin 4B-RING E3 ligase and polycomb repressive complex 2 epigenetic pathway impairs glucose homeostasis^10^. Collectively, these data indicate an essential role of SST-secreting cells in maintaining blood glucose homeostasis. However, studies involving *Sst* gene knockout mice have cast doubt on the functional importance of SST-producing cells, as these mice display normal growth curves, resting normoglycemia and insulin sensitivity compared with age-matched wild-type mice^7, 11,14^. Therefore, the functional roles of SST-producing cells deserve a more explicit investigation.

**Fig. 4:**
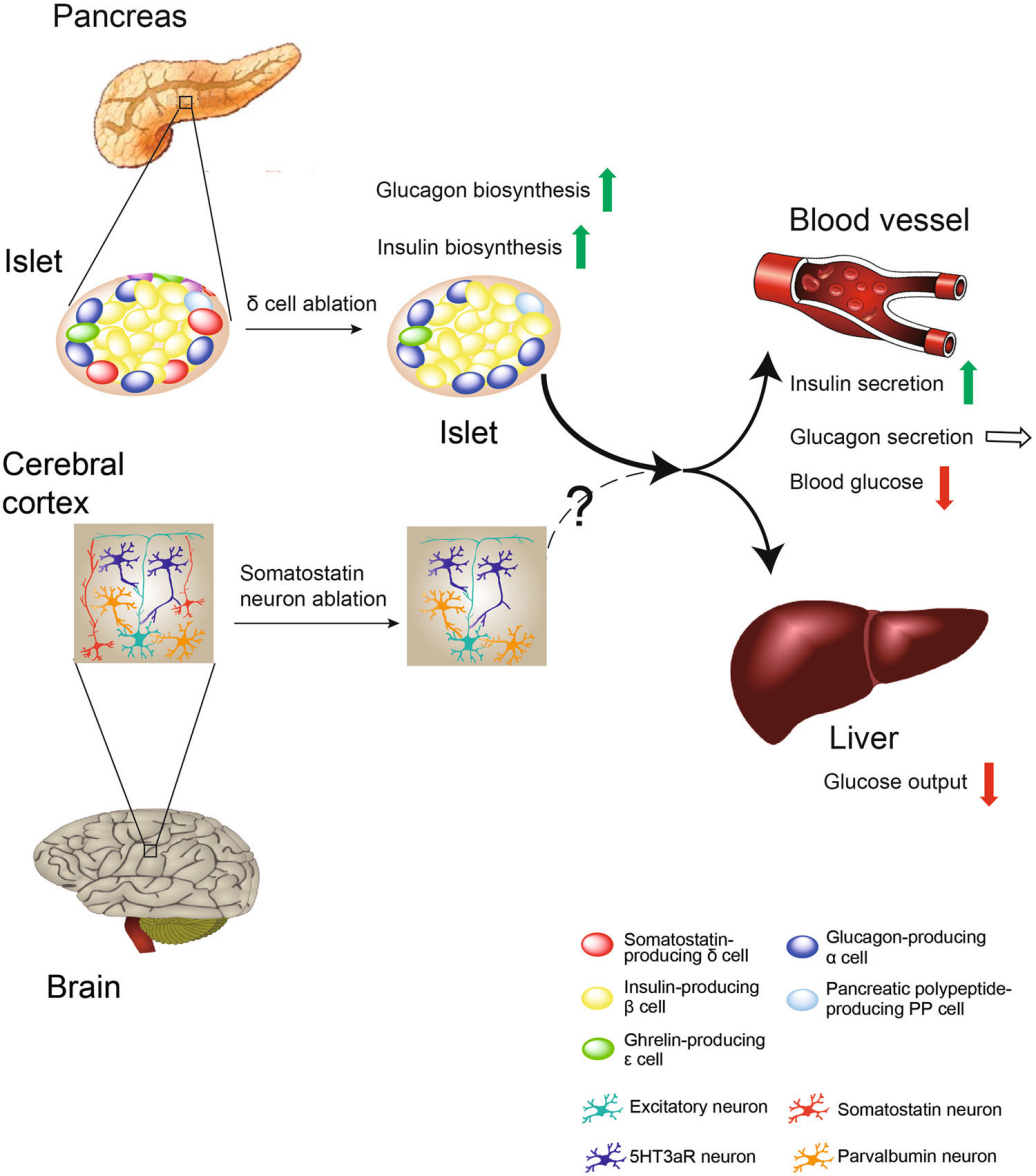
Graphic model of the SST cell ablation-induced dysregulation of glucose homeostasis and neonatal death in mice. Using the Cre-loxP system, the somatostatin-producing cells in mice are specifically ablated. The pancreatic SST cell ablation leads to the proportional increase of other hormone-producing cells within the islet, including glucagon-producing α cells, insulin-producing β cells and pancreatic polypeptide-producing pp cells, without altering the total islet cell content. The resultant excessive synthesis and release of insulin, which serves as the only blood glucose-lowering hormone, lead to severe hypoglycemia and neonatal death of mice. The life expectancy of SST cell-deficient mice could be increased to a limited extent by glucose treatment. These results indicate the essential role of SST-secreting cells in the functional regulation of islet circuit and in the maintenance of glucose homeostasis. However, the potential contribution of somatostatin-secreting cell from other tissues, such as the cerebral cortex where somatostain neurons represent approximately 30% of the total interneuron population, could not be determined in the present study and still await further investigation using tissue-specific SST cell knockout strategy

In the present study, we observed that selective ablation of SST cells caused neonatal death and impaired blood glucose homeostasis (Fig. 4). Our results provide direct evidence highlighting the functional importance of SST-producing cells. Our mechanistic analyses revealed that SST cells regulate resting blood glucose in neonatal mammals and control insulin levels. Interestingly, this regulation of insulin release by SST-positive δ cells in pancreatic islets was found to be mediated by CRHR2 but independent of SSTR, suggesting the secretion of glucose-regulating factors other than SST produced by pancreatic SST-secreting cells via CRHR2 activity. Indeed, although SST is the predominant hormone produced by pancreatic SST-secreting δ cells, moderate expression of other peptides, such as islet amyloid polypeptide (IAPP or amylin) and peptide YY, have also been identified in δ cells^16–18^. Both IAPP and peptide YY have been reported to be functionally involved in the regulation of glycemic control^19,20^. Intriguingly, *IAPP* knockout mice show increased insulin release and faster glucose elimination, which partially phenocopy *Sst*-deficient mice^21^. Moreover, *Pyy* knockout mice exhibit hyperinsulinemia in both fasted and fed states compared with wild-type mice^22^. These results imply an inhibitory tone exerted by IAPP and PYY on insulin release. Therefore, it will be of interest to investigate whether these hormones secreted by SST cells function synergistically with SST in regulating β-cell activities.

Although the pancreatic glucagon mRNA level and glucagon content of the *Sst*^*Cre*^ *R26*^*DTA*^ mice were significantly higher than those of their *Sst-cre* littermates, the plasma glucagon level of these mice were comparable (Fig. 2d). The lack of glucagon release in response to hypoglycemic conditions of the *Sst*^*Cre*^ *R26*^*DTA*^ mice is likely due to the excessive insulin synthesis and release, because insulin has been demonstrated to be a negative regulator of glucagon secretion from α cells. The activation of insulin receptors on α cells decreases the K_ATP_ channel sensitivity to ATP inhibition and promotes the type A GABA receptors translocation, which lead to an increase in the efflux of K^+^ and the influx of Cl^−^, respectively, contributing to the hyperpolarization of α-cell membrane and the resultant suppression of glucagon secretion^23–25^. In addition to glucagon, the counter regulatory responses to insulin-induced hypoglycemia include the increase of various hormones, such as glucocorticoids and catecholamines. Hypoglycemia is a potent activator of the HPA axis and glucocorticoids has been documented to increases glycogenolysis and gluconeogenesis^26–29^. Accordingly, we observed significant elevation of both plasma corticosterone and ACTH levels in *Sst*^*Cre*^ *R26*^*DTA*^ mice. However, the plasma epinephrine level in *Sst*^*Cre*^ *R26*^*DTA*^ mice was slightly but not significantly decreased compared with their *Sst-cre* littermates. This result is intriguing since previous studies have revealed an inhibitory effects of brain SST signaling on stress-related sympathetic activation^30,31^. Therefore, the potential correlation between SST-positive cells and sympathoadrenal system still await further investigation. Nevertheless, our results suggested that the blunted responses of glucagon and epinephrine participated in the deterioration of the hypoglycemia in *Sst*^*Cre*^ *R26*^*DTA*^ mice.

Congenital hyperinsulinism (CHI), which is the most common cause of persistent hypoglycemia in early infancy, can cause permanent brain damage under inappropriate treatment^32^. CHI usually results from genetic defects that either affect all cells of the pancreatic islets (diffuse CHI) or form a focal lesion (focal CHI). Atypical CHI, which is without genetic cause and features morphological mosaicism of islets, has also been reported^33^. Notably, pancreatic δ cells have been shown to be associated with the pathogenesis of all types of CHI. For example, the ratio of β to δ cells in infants with diffuse CHI was demonstrated to be significantly higher than in normoglycemic age-matched controls^34^. Moreover, in patients with either diffuse or atypical CHI, SST was found to be highly co-expressed with NKX2.2, which is a marker of prenatal immature δ cells, indicating functional abnormality of these δ cells^35,36^. Accordingly, our results show that SST cell deficiency in neonatal mice lead to proportional changes of pancreatic β cells, contributing to persistent insulin synthesis and release. Therefore, our findings are of clinical significance and further support the notion that CHI is an islet-based problem rather than being singularly caused by β-cell dysfunction. Currently, SST analogs, such as octreotide and lanreotide, are employed as useful clinical agents for treating diazoxide-unresponsive CHI patients. SST analogs were known to inhibit insulin release through activation of both ATP-sensitive K^+^ channels and G protein-coupled inward rectifier K^+^ channels^37^. It was also reported that SST decreases insulin release through direct interactions with the exocytotic machinery in a pertussis toxin-sensitive mechanism^38,39^. However, SST analog therapy is generally associated with multiple adverse effects, such as cholelithiasis, enterocolitis, and growth retardation, due to the wide distribution of SST receptors in numerous tissues^32,40^. Our results suggest that novel therapies directly reviving pancreatic δ cell activity might achieve better therapeutic effects for certain cases of CHI.

Finally, it is also worth noting that the insulin-releasing mechanisms are not exactly the same in human and rodent models^41,42^. For example, glucose-stimulated insulin release is principally regulated by SSTR5 in rodent β cells whereas the SST effects are predominantly mediated by SSTR2 in human β cells^43,44^. Therefore, the functional mechanism by which SST-secreting cells regulate basal insulin release proposed in the current study using rodent models needs further evaluation in human islets.

In conclusion, we found that the ablation of SST-secreting cells in mice leads to neonatal death and the dysregulation of basal pancreatic insulin synthesis and release. We further demonstrated that SST-positive cells in pancreatic islets regulate basal insulin release in a CRHR2-mediated, but SSTR-independent manner.

## Materials and methods

### Animals

The ROSA26-eGFP-DTA mice (ROSA26^eGFP-DTA^, stock number 006331) was purchased from Jackson Laboratory and Ssttm 2.1 (cre) mice with a congenic C57BL/6 background were generated by crossing Ssttm 2.1 (cre) mice with C57BL/6 mice as described previously^10^. Mice were housed at 22 °C–24 °C on a 12-h light/12-h dark cycle with ad libitum access to water and standardized chow diet (Beijing Ke’ao Xieli Animal Feeds Co. Ltd, Beijing, China). Rats of Wistar strain were housed and bred under specific-pathogen-free conditions at Shandong University animals care facility. For most experiments, neonatal mice or rats were used. Animal experiments included data from both male and female animals. The numbers of animals studied per genotype are indicated in the figure legends within each experiment. All animal care and experiments were reviewed and approved by the Animal Use Committee of Shandong University School of Medicine.

### Glucose rescue experiment

Administration of 50 μl of 10% glucose (cat. #G8270, Sigma-Aldrich) or 50 μl of vehicle control (sterile phosphate-buffered saline (PBS) pH 7.4) was performed by subcutaneous injection into the neonatal *Sst*^*Cre*^ *R26*^*DTA*^ mice at birth and every 8 h thereafter. The survival time and rate of mice were recorded.

### Plasma and pancreatic hormones measurements

For plasma insulin, glucagon, SST, epinephrine, corticosterone, or ACTH levels determination, blood was collected from neonatal rats (postnatal day 2) treated with cyclosomatostatin, Ast2B or vehicle only for indicated times, or from neonatal mice by decapitation. Insulin levels were measured by using an ultrasensitive mouse insulin ELISA kit (cat. #EZRMI-13K, Millipore) and were described as previous^45,46^. Glucagon levels were detected by using the Rat/Mouse glucagon kit (cat. #EZGLU-30K, EZGLU-30BK, Millipore). SST levels were detected by using the Rat/Mouse glucagon kit (cat. #EK-060-03, Phoenix Pharmaceuticals). Epinephrine levels were detected using the Mouse epinephrine kit (cat. #JL11194, Jianglaibio). Corticosterone levels were detected using the R&D corticosterone kit (cat. #KGE009, R&D Systems). ACTH levels were detected using the Mouse ACTH kit (cat. #JL12373, Jianglaibio).

To calculate the insulin or glucagon content of the whole pancreas, the pancreatic tissues isolated from 9- to 12-h-old neonatal mice were homogenized with 500 μl cold acid ethanol solution (75% v/v ETOH and 1.5% v/v conc. HCl). After overnight incubation at 4 °C, the supernatants were collected by 12,000 rpm centrifugation and were neutralized by 1 m Tris (pH 7.5). The insulin and glucagon levels were measured with the ELISA kits following the manufacturer’s instructions. Both insulin and glucagon contents were normalized to pancreas weight.

### Immunofluorescence study

After overnight fixation in 4% paraformaldehyde at 4 °C, the pancreases dissected from neonatal mice were putted in 10% sucrose for 4 h, 20% sucrose for 8 h, and 30% sucrose for 12 h, sequentially. The pancreases slices were first incubated at 4 °C with blocking buffer for 90 min, and then incubated overnight at 4 °C with anti-insulin (cat. #sc-9168, Santa Cruz, 1:250), anti-SST (cat. ab30788, Abcam, 1:500), or anti-glucagon (cat. #AB932, Millipore, 1:250) antibodies. After washing three times with PBS, the slices were incubated at 4 °C for 2.5 h with goat anti-rabbit antibody (cat. #A27034, Invitrogen) or goat anti-rat antibody (cat. #ab150158, Abcam). The slices were stained with 4′, 6′-diamidino-2-phenylindole and subjected to fluorescence microscopic analysis as previously described^47,48^.

### Isolation of pancreatic islets, dispersion, and culture

Neonatal rats were killed by cervical dislocation and pancreata were isolated and digested by collagenase P (cat. #11213865001, Roche) at 37 °C for 10 min. Digestion was stopped by Hank’s balanced salt solution (136.9 mmol/L NaCl; 5.4 mmol/L KCl; 1.3 mmol/L CaCl_2_; 0.8 mmol/L MgSO_4_; 0.44 mmol/L KH_2_PO_4_; 0.34 mmol/L Na_2_HPO_4_; 5.55 mmol/L d-glucose; 4.4 mmol/L NaHCO_3_, pH = 7.4) followed by sedimentation for three times at 4 °C. The islets were collected by hand picking using a stereomicroscope and were cultured overnight in complete media containing 5.56 mM glucose, 10% v/v fetal bovine serum and 0.1% v/v penicillin/streptomycin. Then islets were hand-picked to different groups according to the requirement of experiments. After 30-min incubation in modified Krebs–Ringer bicarbonate buffer (120 mmol/L NaCl, 5 mmol/L KCl, 2 mmol/L CaCl_2_, 1 mmol/L MgCl_2_, 24 mmol/L NaHCO_3_ and 15 mmol/L HEPES at pH 7.4), the grouped islets were subjected to cyclosomatostatin (cat. #ab141211, Abcam), Ast2B (cat. #2391, Tocris), or Ucn3 (Cat. #S3941, Sigma-Aldrich) stimulation for indicated times.

### Islet insulin and SST measurement

The grouped islets were treated with cyclosomatostatin or Ast2B for indicated times and concentration, and the supernatant fraction was collected to quantify the insulin or SST secretion. For insulin or SST contents measurement, the islets were lysed with 50 μl cold acid ethanol solution as previously described^10,46^. After overnight incubation at 4 °C, the supernatants were collected by 12,000 rpm centrifugation and were neutralized by 1 m Tris (pH 7.5). The insulin and SST levels were measured with the ELISA kits following the manufacturer’s instructions.

### RNA extraction and quantitative real-time PCR

RNA from *Sst-cre* and *Sst*^*Cre*^ *R26*^*DTA*^ mice, as well as islets derived from neonatal rats were extracted with TRIzol reagent (Invitrogen). We performed complementary DNA synthesis using the qRT-PCR Kit (Toyobo, FSQ-101). qRT-PCR was conducted in the LightCycler qPCR apparatus (Bio-Rad) with the FastStart SYBR Green Master (cat. #2147483647, Roche) as previously described^10,48^. All primers used for qRT-PCR assay are listed in Table S1.

### Statistics

The experimental data were analyzed utilizing standard parametric statistics including Student's *t*-test, one-way ANOVA or log-rank test where applicable as indicated in the figure legends. Both male (M) and female (F) mice or rats were used based on availability from the breeding cohorts. Statistical analyses were performed using GraphPad Prism 6 software. Data are expressed as mean ± SEM, and statistical values are represented significant when *p-*values were below 0.05. No randomization or blinding was performed in this study and the study was not pre-registered.

## Electronic supplementary material

Supplemental Materials
