# Supplementary material for: Ablation of somatostatin cells leads to impaired pancreatic islet function and neonatal death in rodents
Source: Cell Death Dis. 2018 Jun 7;9(6):682. doi: 10.1038/s41419-018-0741-4 (PMC5992210; doi:10.1038/s41419-018-0741-4)
Supplement: Supplementary file 1 — Supplemental Materials [file 41419_2018_741_MOESM1_ESM.pdf]

SUPPLEMENTARY Materials

Supplemental Figure 1. Generation of mice with SST positive cell-specific ablation. (**A-B**) Schematic representation of somatostatin-producing cell type-specific DTA expression mice (Sst^{Cre} $R26^{DTA}$ mice); The Cre enzyme, the expression of which was driven by the somatostatin promoter (Sst^{Cre}), recognized the LoxP sequence and cleaved the floxed region that prevented DNA transcription, thus enabling the expression of DTA in Sst^{Cre} $R26^{DTA}$ mice. DTA promotes the ADP-ribosylation of eukaryotic elongation factor 2 (eEF2), which is an essential regulator of protein synthesis that mediates the translocation of ribosome. Consequently, the inactivation of the eEF2 by DTA inhibits the protein synthesis and result in the specific ablation of the somatostatin-producing host cell. (**C**) PCR analysis of genomic DNA from tails.

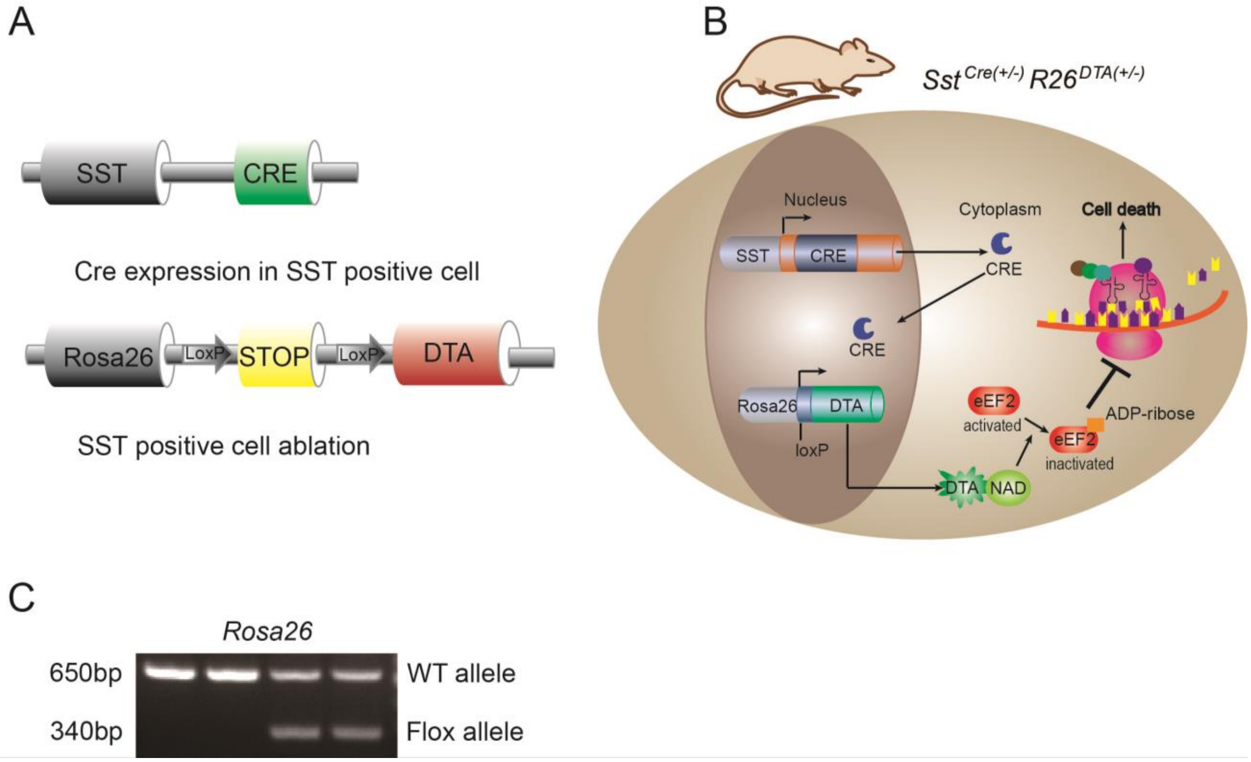

Supplemental Figure 2. Verification of SST positive cell-specific ablation. (**A**) The mRNA levels of SST in the brain of neonatal *Sst*^{Cre} and *Sst*^{Cre} *R26*^{DTA} mice determined by q-RT-PCR (n = 3). (**B**) Immunostaining for somatostatin (SST, red) in stomach sections from neonatal *Sst*^{Cre} and *Sst*^{Cre}*R26*^{DTA} mice (Scare bar: 50 μm). (**C**) Quantification of pancreatic SST positive cell population in the islets of neonatal *Sst*^{Cre} and *Sst*^{Cre}*R26*^{DTA} mice. n = 6 mice per group. 4-6 random areas were selected from each islet section and 5 sections were randomly selected from each mouse. **A-C** ***p<0.00; *Sst*^{Cre} *R26*^{DTA} mice were compared with their *Sst*^{Cre} littermates. Data were shown as mean ± SEM. The data statistics were analyzed using one-way ANOVA.

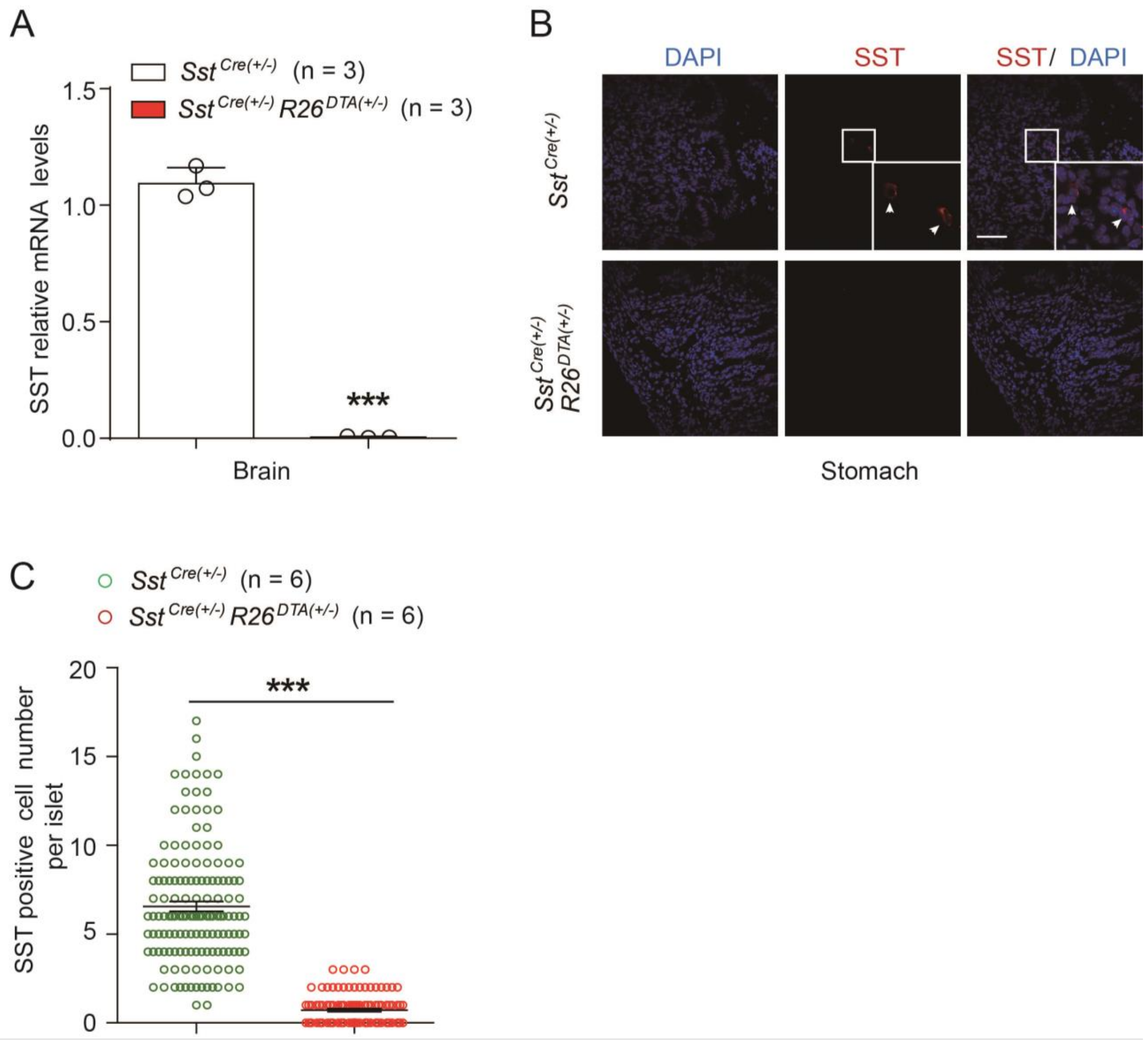

Supplemental Figure 3. Effect of cyclosomatostatin and Ucn3 on islet insulin secretion. (A) Effect of cyclosomatostatin on glucose-stimulated insulin secretion of islets isolated from neonatal rats. **(B)** Effect of Ucn3 on insulin secretion of islets isolated from neonatal rats (Data are from 3 independent experiments. For each experiment, islets from 8-10 neonatal rats were picked and grouped to 50 islets.) **A** **p<0.01; n.s. no significant difference; rats pancreatic islets treated with 12 μM cyclosomatostatin at 1 mM glucose or with vehicle at 11.1 mM glucose were compared with those treated with vehicle at 1 mM glucose. #p<0.05; rats pancreatic islets treated with 12 μM cyclosomatostatin at 11.1 mM glucose were compared with those treated with vehicle at 11.1 mM glucose. **B** **p<0.01; rats pancreatic islets treated with Ucn3 were compared with those treated with vehicle only. Data were shown as mean ± SEM. The data statistics were analyzed using one-way ANOVA.

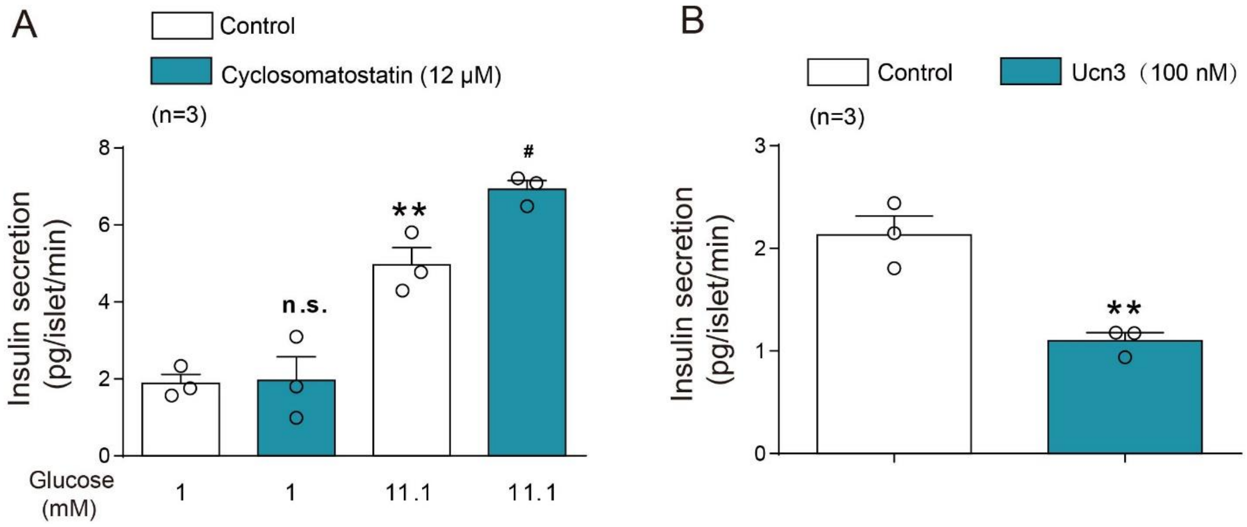

Supplemental Table 1. Primers used for qRT-PCR assay.

PRIMER	POSITION	5'-SEQUENCE-3'
Mus-Insulin-F	Exon1 (1-195)	^{164}CCAGCTATAATCAGAGACCA^{184}
Mus-Insulin-R	Exon2 (314-719)	^{479}GTGTAGAAGAAGCCACGCT^{460}
Mus-Glucagon-F	Exon3 (4938-5099)	^{5002}ACTCACAGGGCACATTCACC^{5022}
Mus-Glucagon-R	Exon5 (7795-7938)	^{7912}CCAGTTGATGAAGTCCCTGG^{7892}
Mus-PP-F	Exon1 (1-35)	^{17}ACTAGCTCAGCACACAGG^{35}
Mus-PP-R	Exon4 (1235-1370)	^{1271}AGACAAGAGAGGCTGCAAGT^{1251}
Mus-Ghrelin-F	Exon3 (754-870)	^{768}AAGAAGCCACCAGCTAAAC^{787}
Mus-Ghrelin-R	Exon4 (2717-2827)	^{2736}ATCGAAGGGAGCATTGAAC^{2717}
Mus-Somatostatin-F	Exon1 (1-232)	^{94}ATGCTGTCCTGCCGTCTCCA^{114}
Mus-Somatostatin-R	Exon2 (897-1259)	^{1109}CTAACAGGATGTGAATGTCTTC CAG^{1084}
Mus-Actin-F	Exon2 (1063-1191)	^{1151}GGCTGTATTCCCCTCCATCG^{1171}
Mus-Actin-R	Exon3 (1279-1392)	^{1392}CCAGTTGGTAACAATGCCATGT^{1370}
Rattus-insulin-F	Exon2 (163-363)	^{176}ATGGCCCGTGGGATCCGCTT^{196}
Rattus-insulin-R	Exon3 (863-1068)	^{918}TGCCAAGGTCTGAAGGTCAC^{898}
Rattus-GAPDH-F	Exon2 (2263-2469)	^{2398}TTCAACGGCACAGTCAAGGC^{2418}
Rattus-GAPDH-R	Exon3 (2550-2640)	^{2636}GCCTTCTCCATGGTGGTGAAG^{2617}
